# An insight into advanced glass systems for radiation shielding applications: A review on different modifiers and heavy metal oxides-based glasses

**DOI:** 10.1016/j.heliyon.2024.e40249

**Published:** 2024-11-08

**Authors:** M.S. Al-Buriahi, Recep Kurtulus, Canel Eke, Sultan Alomairy, I.O. Olarinoye

**Affiliations:** a Department of Physics, Sakarya University, Sakarya, Turkey; b Faculty of Engineering, Department of Materials Science and Engineering, Afyon Kocatepe University, Afyonkarahisar, Turkey; c Akdeniz University, Faculty of Education, Department of Mathematics and Science Education, 07058, Antalya, Turkey; d Department of Physics, College of Science, Taif University, Taif, 21944, Saudi Arabia; e Department of Physics, School of Physical Sciences, Federal University of Technology, Minna, Nigeria

**Keywords:** Glass systems, Modifying oxides, Radiation, Shielding applications, Waste glasses

## Abstract

Ionizing radiation from natural and many synthetic sources is a remarkable tool in many scientific, production, quality control, food preservation, medical, security, and other technological processes. The need to protect humans (public and personnel), gadgets, the environment, and animals from the harmful effects of radiation, while maintaining and expanding the scope of application has made radiation protection an important topic to discuss. Among the methods and materials available for radiation control, shielding and the use of glass shields are the most effective and attractive methods and materials, respectively. In this report, the basic parameters for measuring shielding competences, basic shielding materials and their shortcomings, and glass shields are discussed. Five categories of glasses, namely, borate, germanate, silicate, phosphate, and tellurites, with important shielding attributes, are reviewed. The role of chemical composition, density, and mean atomic number as gamma shielding delineating factors was emphasized. The weaknesses and comparable advantages of each glass system were presented as well. The review concludes by looking at the trend and future of glass shields and research in radiation technology. The data and analysis presented in this review provides scientists and radiation protection technologist on the impact of certain chemical oxides on shielding efficacies of different glass systems.

## 1 Introduction

Ionizing radiation (IR) has seen a tremendous increase in sources and applications in the last century. Science and technology advancements have led to the application of IR in various fields such as medicine, diagnosis and treatment of health trauma, nuclear power plants, nuclear research, biological research, material characterization and modification, and industrial processes [1,2]. Common forms of IR include high-energy photons (x- and gamma-rays), charged particles, including beta (β) particles, protons and heavy ions, and neutrons belonging to different energy spectra. These radiation qualities have enough energy to cause atomic excitation, displacement, ionization, and different other effects in atoms and bulk materials, depending on their energy, dose, and duration of interaction [3,4]. These effects are caused by the transfer of IR energy to the interacting atom or atoms within a bulk material. The energy deposited in a material during radiation interaction can be damaging, if uncontrolled, and beneficial when well managed. Radiotherapy uses the destructive ability of IR to destroy diseased tissues, while tool sterilisation and food preservation processes use it to stop the growth of microorganisms. On the other hand, uncontrolled exposure of materials and living tissues to IR can result in deleterious effects, potentially leading to tissue malfunction or gadget destruction [[5], [6], [7]].

### 1.1 Principles of radiation protection

Different international and local radiation control organisations have established, recommended, and sometimes, imposed radiation protection protocols to mitigate the risk associated with unintended radiation exposures. The cardinal objectives of radiation protection protocols are, to eliminate nonstochastic effects, while reducing the possibility of stochastic effects on radiation users, other radiation facility personnel, the public, and the environment. The International Council of Radiation Protection (ICRP) has recommended three basic principles of radiation protection [[10], [11], [8], [9]]. These are:

(i). justification for the radiation procedure
(ii). optimizing the radiation process and
(iii). dosage limitations.

The justification of practice requires that, in all applications of radiation, the benefits should outweigh the associated risk of radiation exposure. Therefore, the licence, approval, or employment of any practice involving the use of IR should not be granted without conducting a cost-benefit analysis. The optimization process ensures that personnel and public doses are as low as reasonably possible (ALARA). In dose reduction, the ALARA principle takes social and economic factors into consideration, especially for occupational exposures. Finally, all radiation facilities must enforce adherence to dose limits. The dose limits are the maximum doses that different categories of individuals can be exposed to during radiation processes.

Many radiation technologies are justified and processes are optimised to reduce exposure doses within recommended dose limits. In radiation technologies, the management of radiation doses entails the use of three fundamental factors: time, distance, and shielding [7]. The accumulated dose and severity resulting from radiation exposure can be controlled when the time spent using radiation sources is managed. Absorbed doses vary linearly with dose rate and time. Therefore, the absorbed dose from a source with a constant dose rate increases with time. Reducing the time of exposure can reduce the radiation dose. Another effective way to reduce radiation doses is to increase the distance between the source of radiation and individuals. Operating at the highest distance possible from a source of IR is an efficient way to reduce doses to ALARA. The use of a shield is the third factor; it is the most cost-effective, prominent, and practical method of radiation control; it requires the least administrative control. The IR shield is a barrier that confines radiation flux within a volume of space, such that radiation doses outside the confinement are within safe or accepted limits. Different radiation processes necessitate distinct shielding objectives. Therefore, the choice and design of radiation shields vary depending on shielding parameters, dose limits, structural demand, cost, weight, available space, and the nature of incident radiation, among other factors.

### 1.2 Shielding parameters

The assessment of the shielding competence of a medium can be assessed through a host of parameters, depending on the radiation quality.

#### 1.2.1 Photons

**Fig. 1:**
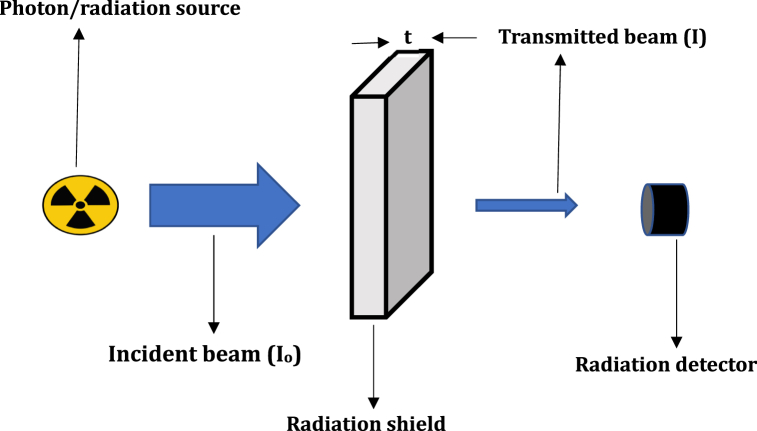
Radiation transmission through a shield of thickness t.

Gamma and x-ray photons are the most popular IR employed in modern technologies. Their widespread applications explains why the shielding efficacy of different materials are tested with respect to photons. Because photons do not possess charge and have zero rest masses, they are non influenced by electric fields created by charged subatomic particles. These enables photons to penetrate deeper into materials, thus a main target for radiation protection protocols. Furthermore, photons can be produced from the interactions of charged particles and neutrons with shielding barriers, consequently, analysing a material for its shielding efficacy against photons is important in radiation science and technology. The linear and mass attenuation coefficients are fundamental photon interaction parameters which can be used to delineate shielding competence among different materials. Fig. 1 shows how μ can be measured. The equation describing the photon transmission process depicted in Fig. 1 is the adjusted Beer-Lambert Equation [8]**:**

(1) $$I\left(E,t\right)=B\left(E,\mu t\right)I_{o}\left(E\right)e^{\mu t}$$

In a narrow beam transmission situation where the beam is narrow, singled energy, an the absorber is thin thus preventing cascade (or buildup) of photons after multiple scattering, the buildup factor B is equal to unity. Hence, μ is determined as [12,13]:

(2) μ=ln(IoI)t

To estimate μ from experimental data, $I_{o}$ and I can be measured as photon intensities, number of photons, exposures, dose rates, or absorbed doses. When t is in cm, μ is in cm^−1^. The linear attenuation coefficient expresses how well a material absorbs photons per unit length. Therefore, a material with a higher μ is less transparent to photons. Aside energy, the linear attenuation coefficient is also sensitive to material density. When μ is normalised for absorber density, the quantity becomes the mass attenuation coefficient (μρ). Other parameters commonly used for measuring photon shielding prowess include, mean free path (λ), half-value layer (HVL), effective atomic number (*Z*_*eff*_), and effective electron density (*N*_*eff*_). These quantities can be estimated directly from μ and μρ as follows [14]:

(3) $$\lambda =\frac{1}{\mu }$$

(4) HVL=ln2μ

(5) Zeff=∑ifiAi(μρ)i∑ifiAiZi(μρ)i

(6) $$N_{eff}=\frac{N_{A}Z_{eff}}{\left\langle A\right\rangle }$$

The weight fraction, atomic number, atomic mass number, and mass attenuation coefficient of the atomic species present in the composite materials are given as $f_{i}$, $Z_{i}$, $A_{i}$, and (μρ)i, respectively. The value of μρ can be obtained directly from experimental data, simulation data using Monte Carlo codes such as FLUKA [15], Geant4 [16], MCNP [17], and PHITS [18] or directly direct computation database such as XCOM [19], WinXCOM [20], Phy-X/PSD [21], Epixs [22], Phy-X/ZeXTRa [23], NGCal [24], and Microshield [25]. Research has shown that high density and atomic number positively influenced the attenuation coefficient of an atom. For composite materials, the density and chemical composition are major factors influencing the gamma absorption prowess.

#### 1.2.2 Light and heavy ions

The interactions of charged radiation leading to attenuation are described mainly by the stopping powers and projected range of the particles in a medium. The mass stopping power $S_{p}$ of electrons and heavy ions are given in Equation (7) and **(8),** respectively [26].

(7) $$S_{p}=4\pi r_{o}^{2}z^{2}\frac{mc^{2}}{\beta^{2}}NZ\left[\ln\left(\frac{\beta \gamma \sqrt{\gamma -1}mc^{2}}{I}\right)+\frac{1}{2\gamma^{2}}\left[\frac{{\left(\gamma -1\right)}^{2}}{8}+1-\left(\gamma^{2}+2\gamma -1\right)\ln\;2\right]\right]$$

(8) $$S_{p}=4\pi r_{o}^{2}z^{2}\frac{mc^{2}}{\beta^{2}}NZ\left[\ln\left(\frac{2mc^{2}}{I}\beta^{2}\gamma^{2}\right)-\beta^{2}\right]$$

These equations shows how the radiation and attenuating material's properties influence $S_{p}$. Comparatively, materials with lower linear stopping powers are more transparent to charged radiation and the radiation have higher range in such absorbers. Stopping powers and ranges of particles could be obtained theoretically using the Monte Carlo simulation code, SRIM [27] and NIST database (ESTAR, PSTAR, ASTAR) [28] using the chemical makeup to describe the absorber. Similar to photons, density and chemical content are the main factors dictating the trend of stopping powers and range of charged radiation.

#### 1.2.3 Neutrons

Unlike photons, the interaction of neutrons changes as the energy spectrum changes. Therefore neutrons are classified based on their energies. Shielding calculations for neutrons involved mainly two classes of neutrons; thermal and fast neutrons. The interaction an shielding competence of materials are measured using interaction cross-sections. The fast neutron removal macroscopic cross-sections ($\Sigma_{R}$) and total microscopic cross-section for thermal neutrons ($\sigma_{tot}$) describe the interaction probabilities. Therefore higher cross-section is an indication higher interaction and collision probabilities. The partial density described by (9), (10), (11) can be used to theoretically estimate $\Sigma_{R}$ [[29], [30], [31]].

(9) $$\Sigma_{R}\left({cm}^{-1}\right)=\sum {\rho_{i}\times \left(\frac{\Sigma_{R}}{\rho }\right)}_{i}$$

(10) $$\rho_{i}=f_{i}\rho$$

(11) $${\left(\frac{\Sigma_{R}}{\rho }\right)}_{i}=\left\{ \begin{array}{c} 0.19Z^{-0.743},for\;Z_{i}\leq 8\\ 0.125Z^{-0.565},for\;Z_{i}>8 \end{array}\right.$$

where, $\rho_{i}\;\text{and}\;{\left(\frac{\Sigma_{R}}{\rho }\right)}_{i}$ is the partial density and the FN mass removal cross-section of individual atomic kinds in the absorber.

For thermal neutrons, $\sigma_{tot}$ can be calculated as [29]:

(12) $$\Sigma_{tot}=\sum \sigma_{j}$$

For a compound, total thermal neutron cross-section (CS) can be estimated as [29,31]:

(13) $$\Sigma \;\left({cm}^{-1}\right)=\frac{0.602\rho }{M}\sum n_{i}\sigma_{i}$$

where, $\sigma_{j}\;\left({cm}^{-1}\right)$ represents the absorption, coherent, and incoherent cross-section, respectively. The average molecular weight of the compound and weight fractions of each atomic species in the glasses are specified by M and $n_{i}$, respectively.

Generally, for a given neutron flux (ϕ) interacting with a target volume (V) of a material having macroscopic cross-section (Σ), the reaction or interaction rate (RR) is defined as [32]:

(14) RR=ΣϕV

The macroscopic cross-section, thus measure how well a medium interact or react with neutrons of specific energy.

Monte Carlo simulation codes and software such as MRCsC [33], Parshield [34], NXcom [35], and NGCal [24] have been used to estimate the shielding efficacies of different materials against neutrons theoretically.

### 1.3 Common shielding materials

Radiation shielding efficiency is not the same for all materials. This is because the factors responsible for high interaction coefficients for each radiation types are not always the same. Thus, the choice of radiation shields depend on the nature of radiation and other important physical, economic, structural, environmental, and social factors. A lot of studies have been dedicated to the understanding of how radiation interact with matter and the use of interaction parameters to evaluate radiation shielding competence. In the last decade, research on shielding materials has continued to grow [36] due to the expansion in the use of radiation, the demand for novel materials as shields in special radiation shielding environments, and also to address the draw backs of existing shielding materials. Generally, functional synthetic IR shielding materials (in bulk form) can be categorised into five basic groups, namely, Pb and Pb-based materials, concrete, polymers, non-Pb metals and alloys, and ceramics and glasses.

#### 1.3.1 Pb and Pb-based materials

Lead and lead-based materials have been the basic shielding materials for gamma and x-rays since the early days of photon application in medicine. With a high density of about 11.34 g/cm^3^ and an atomic number of 82 which translates to high photon cross-section. This makes Pb-based materials attractive for shielding applications. In addition, Pb has good physical and mechanical attributes that make it a desirable and effective field in many radiation processes. Despite its success as a pioneer shielding material in the medical and other applications of radiation, the use of lead may no longer be attractive from an environmental and human health points of views. Lead is a toxic element with the capacity to cause hazards in the human biological system [37,38]. Pb flakes and particles are often formed and fall off the surface of Pb blocks [39]. This constitutes an environmental pollutant that could result in health challenges related to the skin, reproductive, haematological, cardiovascular, respiratory, and nervous systems [[38], [39], [40], [41], [42], [43], [44], [45]]. The fact that Pb bioaccumulates in the human system [39] makes the consequences of Pb-exposure deadly and last for a long time. These factors make Pb an unwanted material in work environments, including radiation facilities. Therefore, the complete elimination of Pb in shielding applications is encouraged, except where the possibilities of Pb flakes fallen off the shields to contaminate the immediate environment can be guaranteed.

In addition, Pb is bulky, dense, and difficult to configure into desired shapes without the fear of releasing Pb dust into the environment. The opaque nature of Pb is another factor that has limited the use of Pb in modern radiation facilities. Though there have been suggestions of cladding Pb blocks with materials such as polymers, thin layer of non-toxic metals such as Al or embedding Pb in chemically stable materials such as glasses and ceramics to boost shielding efficacy and mitigate Pb release in the environment [39]. However, research into the stability, resistance to lead leaching, and Pb-flakes production of the composite materials is still scarce. Also, stability of the coats against radiation damage, temperature changes, and other extreme environmental factors that could be encountered in different radiation environments has not been investigated in detail in the research community. Although Pb-based materials are effective against ions and gamma radiation, alternative Pb-free materials are thus more attractive in contemporary shielding designs from environmental safety perspective. Hence, future research would focus on the environmental safety and stability of Pb-based shielding materials.

#### 1.3.2 Radiation shielding concrete

Concrete is a traditional shielding materials due to low cost of production, composite nature, easy workability, and durability. The success of concrete as a structural shielding material arises from the fact that dense material could be added to improve the shielding efficacy. Also, the inclusion of low and high-Z additives during production can optimise its efficacy to attenuate photons, ions, and neutrons. Thus many concrete flavours have been researched with the aim of producing more effective concrete shields [[46], [47], [48], [49], [50]].

As a shielding material, concrete has many drawbacks, including opacity, chemical instability due to water content variations, presence of pores, structural homogeneity, and cracking due to shrinkage [48]. In addition, the cost of concrete production on the environment is high. Concrete is basically a composite material consisting of a binder (cement), (fine and coarse) aggregates, water. These raw materials are cheaper than many conventional shielding substances. However, the manufacturing of cement considerably increases the emission of CO_2_, a greenhouse gas, and thus causes global warming [51,52]. The manufacturing of cement also results in the emission of dust and toxic chemicals into the atmosphere [53]. The collection of aggregates for concrete making is also associated with ecological issues [54]. These factors make the use of concrete undesirable in some shielding designs. However, the high compressive strength, compositional and geometry flexibility, production simplicity, and financial cost of concrete have made concrete the most popular shielding material per unit volume for a long time. Some of the drawbacks of concrete have continuously been addressed in recent studies [[55], [56], [57], [58]]. The use of more environmentally friendly materials such as geopolymers as replacement for cement has the potential to mitigate the environmental issues related to cement production [55]. The use of geopolymers have also been explored to address the porosity, fire and radiation resistance, thermal and chemical stability issues related ordinary Portland cement-based concrete [[55], [56], [57], [58]]. While the opacity of concrete shields still remains an issue, concrete is still popular as a shielding material where optical transparency is not a requirement. In fact, concrete is the most used civil engineering material in nuclear facilities such as nuclear power stations [[59], [60], [61]]**.** They are used as containment structure, biological, instrument, and thermal shields in nuclear power plants among other important structural functions. Research on concrete shields will continue to grow, especially on improving shielding efficacy, stability, and environmental friendliness.

#### 1.3.3 Polymer and polymer composites

Polymers have great potential to become effective lead-free shielding materials. As composite materials, their components can be chosen to produce remarkable physical and chemical attributes that are superior to those of individual constituents [39,62,63]. Polymer composites are usually light, non-toxic, and thus ecologically safe [64]. These attributes, in addition to their relative low-cost, flexibility, and moderate mechanical strength, make them functional as radiation shields. Although their radiation absorption efficiencies are lower compared to lead, they can be compensated by increasing the thickness of the polymer in shielding designs. Polymers are usually low-weight and contain low-Z atoms; they are thus more effective for neutron-absorbing roles. Doping polymers with materials having a high radiation interaction cross-section is a method of elevating the gamma radiation shielding performance of different polymers. The addition of environmentally safe atoms or compounds with high radiation absorption capacity into polymer matrices has been proven to produce excellent radiation shields. Recently, high-density polyethylene (HDPE) doped with MoS_2_, W, and B_4_C, Bi_2_O_3_-filled poly (methyl methacrylate) composites, nanosized tungsten oxide (WO_3_) doped emulsion polyvinyl chloride (EPVC) polymer composites, lead oxide-filled isophthalic resin polymer composites, silicon rubber composites containing bismuth, polymer bricks (PolyBiz), polyester composites strengthened with zinc, and composites of polyethylene with cadmium oxide, lead oxide, and zinc oxide have been shown to be good radiation absorbers [[64], [65], [66], [67], [68], [69], [70], [71], [72], [73], [74]]. The use of polymers as shields may be restricted due to their lower mechanical strength and thermal resistance compared to other categories of shielding materials.

#### 1.3.4 Metals and alloys

Studies have demonstrated the importance of certain metals as shielding materials. Non-poisonous heavy metals, such as Ba, Bi, and W, have high gamma shielding capacities and are good replacements for poisonous Pb. However, the use of metals in their pure state for shielding applications is not common due to issues related to cost and stability in their physical, chemical, thermal, and mechanical properties. For instance, the high cost of W discourages its use in its pure state [75]. The high cost of Pb has also led to the use of iron (Fe) as cladding for Pb shields in the past [76]. The chemical stability of Fe is a factor that could have limited the expansion of such practices. In addition, aluminum is also known as a good structural material for space radiation shielding [77]. Its mechanical strength and thermal response may have limited its general application as a shield. Consequently, it a common practice to embed metals in glass, ceramics, polymers, and other matrices, use them as alloys, or combine them with other non-metallic materials to mitigate their respective drawbacks and improve their radiation-protective functionality.

Alloys are useful engineering materials formed by combining metals with other metals, metalloids, or non-metals. Alloys are stable and possess interesting attributes that are often different and superior to the base materials. Many times, alloying is encouraged due to the need to compensate for the limitations of the constituent elements and obtain desired features. For instance, using modifiers such as carbides and borides during casting enhanced the strength and heat resistance of Ti-based alloys [78]. Furthermore, brass is a well-known alloy of Cu and Zn with better features compared to the two base metals [79]. Presently, there are more than 100 elements in the periodic table, of which more than 80 are metals. As a result, the spectrum of alloy formation is very wide due to the different possible combinations of metals, metalloids, and non-metals to form stable alloys. Diverse factors such as base-metal composition, application, size, and fabrication technique have been used to classify the large array of available and possible alloys [79].

In radiation shielding, alloys are attractive due to their physical and structural features. Furthermore, one can tailor an alloy's composition to foster the development of high radiation shielding attributes. Researchers have investigated and recommended many alloys for shielding applications, including Cu-, Ni-, W-, Pb-, and Fe-based alloys [[79], [80], [81], [82], [83], [84], [85], [86]]. Many alloys have shown good radiation protection qualities; however, alloy are not universal shields suitable for all radiation protection purposes due to their opaque nature.

Metals are precious materials in many industrial applications, and their non-uniform distributions make them scarce materials. This makes metals and alloys expensive, thus limiting their widespread applications in shielding technology. Fabricating alloys is not always cheap; some effective alloy shields have high density and low workability, making them difficult to form into the required shielding geometry. These factors limit the applications of alloys in radiation protection functions and encourage further research into alloys and alternative materials for radiation control purposes.

#### 1.3.5 Ceramics and glasses

Ceramics and glasses are gradually becoming versatile materials in science and technological processes. The broadband applications results in their ability to serve or function well where polymers, metals, and alloys fail. Ceramics are solid composite of compounds of metal and non-mental atoms. Ceramics are known to have high mechanical strength, corrosive resistance, high thermal stability, and durability. They have thus been shown to be effective shielding materials [[87], [88], [89], [90], [91]]. However they are not optically transparent and not always easy to fabricate. On the other hand, glasses are preferred alternative to other classes of shields due to their physical, mechanical, and optical properties. The fact that glasses can be modified through chemical compositional variations and synthesis techniques makes glasses important materials for many functions including radiation absorption. Diverse glasses are currently utilized for different radiation protection structures globally. Recently, various glasses such as silicate, borate, phosphate, germanate, arsenate, tellurite etc. have been investigated for their structural, mechanical and radiation shielding properties. The attraction in radiation shielding glasses research and applications are due to their useful properties such as low cost of synthesis, elastic properties, recyclable nature, optical transparency, light weight, non-toxic, radiation resilience, and good host for doping with functional atoms [[91], [92], [93], [94], [95]].

The results obtained from recent studies of some glasses reveal that the amount of glass-forming and modifying oxides in a glass system influences the general properties of glasses. For example, Naseer et al. [96] investigated the effect of Bi_2_O_3_ content on the physical, structural, photon, and neutron shielding characteristics of barium telluroborate glasses and reported that enhancing Bi_2_O_3_ content increased the glasses' photon and fast neutron shielding abilities. Researchers recommended the glasses for radiation protection in medical, industrial, and nuclear power plants. In their study [97], Kilic et al. looked into the physical, thermal, optical, structural, photon, neutron, and charged particle shielding properties of borotellurite glasses doped with Sm_2_O_3_. They announced that the addition of Sm_2_O_3_ significantly advanced the radiation shielding ability of the studied glasses. Rammah et al. [98] looked into the structure, optical, and radiation shielding properties of vanadium borophosphate glasses that contain ZnO. They found that the glass was better at attenuating radiation as the ZnO content went up. Therefore, the glasses were recommended as suitable for use as semiconductor and radiation shielding applications. Kaky et al. [99] studied the structural, physical, optical, and radiation shielding characteristics of germanate-tellurite glasses with various contents, and their results showed a reduction in the effective atomic number and density of the examined glasses with increased GeO_2_ content. Almuqrin et al. [100] conducted an analysis on the impact of Li_2_O content on the mechanical and radiation shielding capability of TeO_2_-As_2_O3-B_2_O_3_ glasses. They found that the elastic young, shear, longitudinal, and bulk modules of the studied glasses grew with the enhancement of Li ions, while the linear attenuation coefficient decreased when Li ions replaced Te in the glasses. Al-Buriahi et al. [101] investigated the optical and photon shielding abilities of Li_2_O and MoO_3_-doped tellurite glasses, and their findings revealed that radiation shielding features decrease as Li_2_O content increases. Alothman et al. [102] used theoretical and simulation methods to look into how CeO_2_ affects the photon shielding ability of Fe_2_O_3_-P_2_O_5_ glass-ceramic. Their findings showed that the glasses' photon shielding ability got better as the CeO_2_ content went up, but there were no considerable changes in the fast neutron removal cross section of the glasses as the CeO_2_ content changes. According to Alshahrani et al. [103], they looked into the radiation, neutron, and charged particle shielding properties of tellurite glasses, such as Sb_2_O_3_ and V_2_O_5_, using both theoretical and simulation methods. Their findings showed that adding more Sb_2_O_3_ content has a strong positive effect on the shielding of photons and thermal neutrons, but not on the shielding of charged particles and fast neutrons.

Therefore, a careful combination of modifying oxides in glass fabrication is important for the emergence of glasses with suitable optical and radiation shielding applications. The following sections provide detailed information about the radiation shielding properties of various types of glasses found in recent literature. Although, recent review on radiation shielding glasses has been published by Kurtulus [104], the review address few glasses and failed to give insight into other properties of the glasses that makes them attractive for shielding aside their radiation shielding parameters. The review also focused on photon shielding without discussing charged radiation, and neutron shielding efficacy of the glasses. All these shortcoming are addressed in the present review. In addition the gamma and neutron interaction parameters of common glass forming and modifying oxides were estimated and presented as well. This review is critical for understanding the current trend in shielding radiation glass research and applications. It therefore highlights potential glass systems for transparent shielding and other applications.

## 2 Radiation shielding glasses

Quite a number of glasses have been investigated and recommended for radiation protection applications in last few decades. The number of interest in radiation shielding glasses have grown considerably in the last decade [105]. Significant among the categories of glasses that have been studied for their shielding behaviour and their functionality in radiation protection and nuclear waste management are borate, germanate, silicate, phosphate, and tellurite glass systems along with their waste glass products. In the following section, recent research on these glass categories are presented from radiation control and protection perspectives.

### 2.1 Borate glass

Borate (B_2_O_3_) glasses have been receiving great attention due to their unique characteristics in recent times. Although silicate glasses meet the demand of many common glass applications, B_2_O_3_-based glasses are also important class of glasses due to their relatively lower glass forming (circa 260 °C) and melting (∼450 °C) temperatures compared to silicate (1100 °C and 1728 °C, respectively) and other glass systems [[105], [106], [107]]. This makes the production budget of borate glasses lower and the glasses more attractive for sealing applications, especially in electronics. Among the advanced technical uses of B_2_O_3_-based glasses are optical, optoelectronics, electronics, photonics, biomedical, and luminescence applications [104]. Pure B_2_O_3_ glass is mostly useless for many purposes due to its low chemical stability and water dissolution rate (3.89 × 10^−3^ g/cm^2^.min) [108]. The addition of modifying oxides including alkali, alkaline oxides, PbO, etc. have been found to improve the stability and functionality of borate based glasses [105,[108], [109], [110]]. Also, the combination of SiO_2_ and B_2_O_3_ produces thermally and chemically stable borosilicate glasses, with wide industrial applications. Borate glasses have high solubility for rare earth (REMs) transition (TMs) metals, wide glass forming range, and high order optical nonlinearity [105,111]. These and some other interesting attributes make borate glasses have good compositional flexibility. The compatibility of B_2_O_3_ structure with modifying oxides, TMs, and REMs make B_2_O_3_-glasses vital as industrial, optical and radiation shielding materials.

In many radiation shielding designs, considerations are often given to photons and neutrons due to their high penetration abilities. However, their shielding requirements are not the same; while gamma photons require a dense, high-Z material, neutrons require low-Z materials such as B for high interactions and attenuation. The attractiveness of borate glasses for radiation shielding results from the high neutron cross-section of boron and the flexibility of the borate structure to accommodate heavy metal oxides. These combination when optimised can serve as good radiation attenuator for both neutron and gamma radiation. In addition, neutrons can undergo radiative capture by nuclides, or cause the release of energetic light atoms as shown in Equation (15) for ^10^B interaction with thermal neutrons [112]. Hence, a good gamma absorbing nuclide is important in neutron interaction processes.

(15) B510+n→He24(1.47MeV)+Li37(0.84MeV)+γ(0.84MeV)

^10^B has high cross-section for neutrons and with a natural isotopic abundance of 20 %, borate glasses are thus important neutron absorbing materials.

Various type of borate glasses have been studied for radiation shielding implementations in scientific literature. Aloraini et al. [113] investigated photon shielding characteristics of strontium borate tellurite glasses with chemical formular 10SrO-(90-x)B_2_O_3_-xTeO_2_, where x was equal to 40, 45, 50, 55, and 60 mol% using experimental and theoretical methods between 0.356 MeV and 1.333 MeV gamma energies. They reported that the glass that contained the highest TeO_2_ (60 mol%) has the highest gamma radiation shielding characteristics. This is partly due to the increase in glass density and photon interaction cross-section occasioned by the denser TeO_2_ (5.67 g/cm^3^) relative to B_2_O_3_ (2.46 g/cm^3^) and the atomic number of Te (52) relative to B (5). The research shows that a partial replacement of B_2_O_3_ with TeO_2_ in a borate glass structure increases gamma absorption ability. Yonphan et al. [114] examined gamma-ray interactions and build-up factors of gadolinium sodium borate glass having the chemical formula xGd_2_O_3_: 20Na_2_O:(80-x)B_2_O_3_ where x is 0, 5, 10, 15, and 20 mol% using experimental (0.223 MeV–0.662 MeV) and theoretical (0.015–15 MeV) methods and they noted that substitution of B_2_O_3_ by Gd_2_O_3_ enhance the mass attenuation coefficient (MAC), effective atomic number (Z_eff_), effective electron density (N_eff_). Kavaz et al. [115] investigated the structure and photon shielding capability of bauxite-ore-doped lithium borate with composition (Li_2_B_4_O_7_)_(100-x)(_Bauxite)_x_ where x = 0,10, 20, 30 and 40 % glasses using experimental, theoretical and simulation approach between 81 keV and 283 keV energies and their results indicated that 40 % percent bauxite containing glass posses the best photon and neutron shielding ability of the composite. The presence of Fe, Ti and Al in the ore enhanced the shielding ability of the lithium borate glasses while Li and B influenced the neutron interaction capacity. Eke [116] studied the gamma photon shielding characteristics of 60B_2_O_3_-9ZnO-(30-x)Al_2_O_3_-xBi_2_O_3_-1Sm_2_O_3_ where x = 5, 10, 15 and 20 mol% glasses using theoretical method between 0.015 MeV and 15 MeV and the result showed that BZnAlBiSm-4 which contains higher content of Bi_2_O_3_ has the superior radiation shielding features but BZnAlBiSm-1 which has the smallest Bi_2_O_3_ content has the highest fast neutron removal cross section. Therefore increasing the Bi_2_O_3_ content improved the gamma interaction probability while the lower atomic number Al and O atoms enhanced the ability of the glass system to interact with fast neutrons. The photon shielding features of (Tl_2_O_3_)30-(Li_2_O)10-(B_2_O_3_)(60−y)-(Sm_2_O_3_)y glasses with different Sm_2_O_3_ contents (y = 0, 0.2, 0.4, 0.6) were investigated using theoretical and simulation methods between 0.015 MeV and 15 MeV energies by Issa et al. [117] and their results demonstrated that samarium (III) oxide enhanced radiation shielding properties of studied glasses. Uosif et al. [118] explored the photon shielding ability of tungsten lithium borate glasses with chemical composition (25-x)Li_2_O-75B_2_O_3_-xWO_3_ (x = 1, 3, 5 and 7.5 mol%) using experimental and theoretical methods and they announced that the MACs, Z_eff_ values ascended as WO_3_ content enhances while half value layer (HVL), mean free path (MFP) EBF and EABF descended ad WO_3_ content increased. Kaky et al. [119] examined radiation shielding ability of (80-x)B_2_O_3_–10ZnO–10MgO-xBi_2_O_3_ where x = 10, 20, 30, 40, 50 and 60 mol% glasses using experimental and theoretical techniques and their results displayed that S6 which has 60 mol% of Bi_2_O_3_ has the highest attenuation properties and studied glasses can be used as shielding materials particulary for low photon energies. Sayyed et al. [120] studied radiation shielding properties of (40+x)PbO–5TeO_2_–15BaO–(20−x)Na_2_O–20B_2_O_3_ (x = 0, 5, 10, 15, and 20 mol%) glasses using theoretical approach and simulation code and they reported that MACs of the studied glasses rose with increment of PbO concentration while they declined with increment of photon energy. Alzahrani et al. [121] examined photon, neutron and charged particles shielding characteristics of various content of PbO–B_2_O_3_–Bi_2_O_3_–ZnO glasses using theoretical and simulation method and they pointed out that substitution of PbO with Bi_2_O_3_ slightly declined the photon shielding ability of the studied glasses but reduced the toxicity effect of PbO. Al-Buriahi et al. [122] researched the impact of CdO on the photon, neutron and electron attenaution features of boro tellurite glasses with chemical formula 50B_2_O_3_ - (50-x)TeO_2_- xCdO, where x = 0, 10, 20, 30, 40 and 50 mol% using theoretical and simulation techniques and they explained that CdO has a little effect on the photon, neutron and electron shielding ability of the boro tellurite glasses. Radiation shielding competence of 22SiO_2_-23Bi_2_O_3_-37B_2_O_3_-13TiO_2_-(5-x)LiF- xBaO glasses where x = 0, 1, 2, 3 and 5 mol % studied by Al-Baradi et al. [123] and their results demonstrated that BaO content enhanced the radiation shielding competence. Shaaban et al. [124] theoretically probed the influence of TiO_2_ on the radiation shielding characteristics of 59B_2_O_3_-29SiO_2_-2LiF-(10 − x)ZnO-xTiO_2_ where x = 0, 2, 4, 6, 8, and 10 mol % glasses and they clarified that gamma radiation and fast neutron attenuation properties increased as TiO_2_ content rose. Al-Buriahi et al. [125] examined the effect of WO_3_ on the photon shielding ability of a (55B_2_O_3_–15SiO_2_–30Na_2_O: xWO_3_ where x = 0.0, 0.5, 1.0 and 1.5 wt%) using theoretical and simulation method and they reported that the shielding ability of the studied glasses can be controlled by modifying the content of the WO_3_. Abouhaswa et al. [126] investigated the effect of Sb_2_O_3_ on the photon, neutron, electron and proton shielding features of (60-x)B_2_O_3_- 20Bi_2_O_3_- 20Na_2_O_2_- xSb_2_O_3_ glasses where x = 0, 2.5, 5, 7.5, 10, 15 wt% using theoretical and simulation method and their results indicated that increasing the Sb_2_O_3_ content had positive effects in enhancing the density and shielding capability for all types of radiation. Alothman et al. [127] analyzed the effect of MoO_3_ on the photon shielding features of 55B_2_O_3_–30Pb_3_O_4_–(15-x)Al_2_O_3_-xMoO_3_, (0 ≤ x ≤ 5) and they announced that the highest photon and fast neutron attenuation properties of the glass possessed the most MoO_3_ concentration. Mhareb et al. [128] researched the photon, proton, neutron and alpha shielding properties of (80-x)B_2_O_3_–10SiO_2_–10TiO_2_-(x)BaO for x = 10, 15, 20, 25 and 30 mol% glasses using theoretical and simulation method and their results indicated that addition of BaO enhanced the gamma shielding properties but decreased the fast neutron removal cross section. In a more recent study [129], Bi was used to improve the gamma shielding features of a lead-tungsten-boron glass system. The result for Bi was superior to that of Al and Sb in the same glass system. Table 1 summarises the glass compositions and how each investigated oxides controlled their radiation attenuation competences.

**Table 1:** Summary of effects of some modifying oxides on some recently investigated borate glass system.

Glass composition	Modifying oxide/material	Oxide concentration range (mol%)	Effect on shielding capacity	Ref.
10SrO-(90-x)B_2_O_3_-xTeO_2_	TeO_2_	40–60	Gamma CS increased	[113]
xGd_2_O_3_: 20Na_2_O:(80-x)B_2_O_3_	Gd_2_O_3_	0–20	Gamma CS increased	[114]
(Li_2_B_4_O_7_)_(100-x)(_Bauxite)_x_	Bauxite	0–40	Gamma and FN CSs increased	[115]
60B_2_O_3_-9ZnO-(30-x)Al_2_O_3_-xBi_2_O_3_-1Sm_2_O_3_	Bi_2_O_3_	5–20	Gamma CS increased; FN CS decreased	[116]
(Tl_2_O_3_)30-(Li_2_O)10-(B_2_O_3_)(60−y)-(Sm_2_O_3_)y	Sm_2_O_3_	0–0.6	Gamma CS increased	[117]
(25-x)Li_2_O-75B_2_O_3_-xWO_3_	WO_3_	1–7.5	Gamma CS increased	[118]
(80-x)B_2_O_3_–10ZnO–10MgO-xBi_2_O_3_	Bi_2_O_3_	10–60	Gamma CS increased	[119]
(40+x)PbO–5TeO_2_–15BaO–(20−x)Na_2_O–20B_2_O_3_	PbO	0–20	Gamma CS increased	[120]
(40-x)PbO–50B_2_O_3_–xBi_2_O_3_–10ZnO	Bi_2_O_3_	1–20	Gamma CS decreased	[121]
50B_2_O_3_ - (50-x)TeO_2_- xCdO	CdO	0–50	Gamma CS decreased sightly at low energies; increased for high photon energies, charged radiation and FN CS increased.	[122]
22SiO_2_-23Bi_2_O_3_-37B_2_O_3_-13TiO_2_-(5-x)LiF- xBaO	BaO	0–5	Gamma CS increased	[123]
59B_2_O_3_-29SiO_2_-2LiF-(10 − x)ZnO-xTiO_2_	TiO_2_	0–10	Gamma and FN CS increased	[124]
(55B_2_O_3_–15SiO_2_–30Na_2_O: xWO_3_	WO_3_	0–1.5 wt%	Gamma CS increased	[125]
(60-x)B_2_O_3_- 20Bi_2_O_3_- 20Na_2_O_2_- xSb_2_O_3_	Sb_2_O_3_	1 - 15 wt%	charged radiation and gamma CS increased	[126]
55B_2_O_3_–30Pb_3_O_4_–(15-x)Al_2_O_3_-xMoO_3_	MoO_3_	0–5	Gamma CS increased	[127]
(80-x)B_2_O_3_–10SiO_2_–10TiO_2_-(x)BaO	BaO	10–30	Gamma CS increased; FN CS decreased	[128]
45B2O3–20ZnO −30BaO-5MO	CaO, TiO_2_, and CuO	5	Gamma CS highest for CuO	[129]
(29.5–0.4x)CaO +10CaF_2_ + (60–0.6x)B_2_O_3_ + xTeO_2_+ 0.5Yb_2_O_3_	TeO_2_	10–54	Gamma CS increased	[130]

Borate glasses have features that make them preferable in many applications, the high cross-section of boron relative to other elements makes boron rich glasses attractive from neutron control perspective. The addition of heavy ions into the matrix of borate glasses improves their gamma shielding and charged radiation absorption potential. In addition the optical transparency of borate glasses (which is a function of the modifying oxides it contains) is another reason for the potential applications as transparent shield. Many borate glasses have been investigated for different aspects, however, future studies would focus on improving the mechanical strength, chemical stability, thermal resilience, and radiation damage resistance of the glass system. This is expected to expand the scope of B_2_O_3_-based glasses functionality for many purposes and for different shielding scenarios.

### 2.2 Germanate glasses

GeO_2_ is a high density (4.25 g/cm3) glass former. This makes GeO_2_-based glasses ideal for gamma absorption roles among other basic glass formers (second only to TeO_2_). Germanate glasses are common in optical applications, such as optical amplifiers, color displays, lasers, and ultrafast devices due to their wide optical transmission windows, high index of refraction, low phonon energy, and good hosts for optically active rare earth oxides [133]. A good choice of modifier can tailor germanate glass properties for other novel applications. Combining GeO_2_ and TeO_2_, two dense glass formers have been shown to increases the chemical and thermal stability of germante/tellurite glasses due to the wide working temperature of GeO_2_ [134]. Such combination could also influence the shielding efficacy of germante glasses.

Many germanate glasses have been studied for their shielding behaviours and their remarkable potentials have been highlighted for radiation protection applications [[135], [136], [137], [138], [139], [140], [141], [142]]. In Table 2, a summary of recent findings on the shielding abilities of germanate glasses is presented. These show that germanate glasses can be good radiation absorbers. The radiation shielding abilities of germanate glasses are optimised using other glass formers or modifiers such as TeO_2_, B_2_O_3_, and Bi_2_O_3_. The addition of atoms possessing high radiation attenuation strength also improves the radiation protection features of the glasses. In Ref. [134], Nd_2_O_3_ was used as a partial replacement for Bi_2_O_3_. There was a slight but inconsequential increase in the gamma absorption cross-section of the glass system. Altering the nature of the chemical unit or the concentration of an existing unit both have the tendency to alter the shielding behavior of a germanate glass system.

**Table 2:** Summary of effects of some modifying oxides on some recently investigated germanate glass system.

Glass composition	Modifying oxide/material	Oxide concentration range (mol%)	Effect on shielding capacity	Ref.
(69.7-x)Bi_2_O_3_ -30GeO_2_ -xNd_2_ O_3_	Nd_2_O_3_	0.3–1.0	Gamma CS increased slightly	[133]
30B_2_O_3_ -40GeO_2_ -(29.75-x)Gd_2_O_3_ -xSm_2_O_3_	Sm_2_O_3_	0.25–1.25	Gamma CS increased slightly	[134]
(80-x)TeO2 -(10+x)Li2O-(10+x)GeO2	TeO_2_, Li_2_O, GeO_2_	0–15	Gamma CS increased slightly	[135]
(75-x) TeO2-xGeO2–12.5ZnO-12.5BaO	TeO_2_	0–20	Gamma CS decreased	[135]
(70-x)TeO2-xGeO2–20ZnO-10Li2O	GeO_2_	5–20	Gamma CS decreased; FN CS increased	[136]
50GeO2-(50-x)PbO-xZnO	ZnO	0–50	Gamma CS decreased; FN CS increased	[137]
*(45-x)Li2O - 55GeO2* *- xZnO*	ZnO	0–25	Gamma CS increased	[138]
(60-x)TeO2-10GeO2-20ZnO-10BaO- xBi2O3	Bi_2_O_3_	2.5–10	Gamma CS increased	[139]
xBi2O3 + (80-x)TeO2 + 10B2O3 + 10GeO2	Bi_2_O_3_	40–60	Gamma CS increased; FN CS decreased	[140]
(90-x)GeO2-xPbO-5Al2O3–5CaO	PbO	0–40	Gamma CS increased.0	[141]
x(Bi2O3)40-x(PbO)60(GeO2)	Bi_2_O_3_	0–40	Gamma CS increased	[142]

The scarcity of pure Ge may limit the deployment of Germanate glasses for radiation control measures, despite their demonstrated shielding proficiency. Germanate glasses will thus be expensive, and producing them in good quantity for the purpose of nuclear radiation and waste control may not be encouraging. This could be the reason for the low patronage of research focusing on the shielding behavior of GeO_2_-based glasses compared to borate, silicate, and tellurite glasses. Many of the studies on germanate glasses focused more on optical applications than radiation protection applications. Germanate glasses are thus not attractive from a cost-implication perspective.

### 2.3 Phosphate glasses

P_2_O_5_ is a basic glass former within the class of B_2_O_3_ and SiO_2_ unlike GeO_2_ and TeO_2_ that are regarded as conditional network formers [143]. Pure P_2_O_5_ glass is hygroscopic, hence, there is limitation in its application. The addition of modifiers has been a technique used to improve the stability and make P_2_O_5_-based glasses more functional. P_2_O_5_-based glasses are unique with respect to their unique attributes. Phosphate glasses have low melting temperatures, that foster cheap fabrication method, high dielectric constant, chemical durability, resistance to crystallization, optical transmission within wide wavelength band, good thermal stability and low phonon energy [143]. To extend the properties of phosphate glasses, modifying oxide and other chemical compounds have been introduced in the phosphate glass structure to improve their radiation interaction capacities.

A series of ternary lead zinc phosphate ((PbO)x(ZnO)60-x(P_2_O_5_)40) glasses was investigated for their gamma attenaution capabilities. The gradual replacement of ZnO with PbO was found to improve glass stability, density, and the mass attenuation coefficients (MACs) within wide gamma shielding energies [144]. The addition of PbO had a positive impact on the gamma protective feature of the glass system and make the glasses comparably better than some conventional shields, but, its toxic nature may make the glass unattractive from an environmental perspective. In another investigation, Al-Yousef et al. [145] prepared xBi_2_O_3_+20CaO+10K_2_O+(30-x)Na_2_O+40P_2_O_5_ glasses for x = 0, 2.5, 5, 7.5, and 10 mol% and declared that Bi_2_O_3_ improved the phosphate glass density and gamma-ray interaction probabilities. Unlike PbO, Bi_2_O_3_ non-toxic and the glasses preferred as non-lethal shields. Rammah et al. [146] demonstrated the impact of BaO on the ability of 50P_2_O_5_ + 30TiO_2_ + (20-x) K_2_O + xBaO glasses to attenuate gamma-ray and moderate FNs. It was concluded that the gamma-rays and FN CSs increased and decreased, respectively as BaO increased with respect to K_2_O content. In an attempt to improve the durability of phosphate glass, B_2_O_3_ can be used [147]. Not long ago, Rammah et al. showed the influence of B_2_O_3_ on the density and gamma absorption prowess of V_2_O_5_- P_2_O_5_ glass system. They gradually doped the glass system with B_2_O_3_ between 0 and 8 mol% and showed that the doping compromised the ability of the glasses to absorb photons. Although the neutron attenuation properties were not investigated, it can be hypothetically stated that B_2_O_3_ addition could have improved FN CS due to the high FN CS of B. The glasses were opaque and therefore useless when optically transparent shields are sought after. The scarcity of V could also prevent the glasses from being used for general shielding purposes despite the high gamma shielding propensity. Other modifying oxides such as La_2_O_3_ [148], Al_2_O_3_ [149], Bi_2_O_3_ [150], Eu_2_O_3_ [151], GeO_2_ [152], and WO_3_ [153] have been used to modify the shielding effectiveness and other attributes of phosphate glasses in recent times (see Table 3 for some recent studies). The choice of modifier in shielding application would depend on the shielding environment and radiation quality. The choice of neutron shield for the future would also consider using atoms that have low probability of activation after neutron absorption. Long term stability and resilience to radiation damage are areas requiring efforts in future research on the use of phosphate glasses for radiation control.

**Table 3:** Summary of effects of some metal oxides on some recently investigated phosphate glass system.

Glass composition	Modifying oxide/material	Oxide concentration range (mol%)	Effect on shielding capacity	Ref.
(PbO)x-(ZnO)60-x-(P_2_O_5_)40	PbO	0–60	Gamma CS increased considerably	[144]
xBi_2_O_3_+20CaO+10K_2_O+(30-x)Na_2_O+40P_2_O_5_	Bi_2_O_3_	0–10	Gamma CS increased saliently	[145]
50P_2_O_5_ + 30TiO_2_ + (20-x) K_2_O + xBaO	BaO	0–20	Gamma CS increased; FN CS decreases	[146]
(100−x)0.5V_2_O_5_-0.5P_2_O_5_-xB_2_O_3_	B_2_O_3_	0–8	Gamma CS increased	[147]
50P_2_O_5_–30Sb_2_O_3_–10CaO–5Al_2_O_3_–5TeO_2_+xLa_2_O_3_	La_2_O_3_	0–5	Gamma and FN CSs increased	[148]
xAl_2_O_3_·(40−x)Ag_2_O·60P_2_O_5_	Al_2_O_3_	0–20	Gamma CS increased	[149]
20Li_2_O–35Li_2_WO_4_–(15-x)TiO_2_–xBi_2_O_3_–30P_2_O_5_	Bi_2_O_3_	0–15	Gamma CS increased	[150]
xEu2O3-(15-x)ZnO-10CaO-35PbO-40P_2_O_5_	Eu_2_O_3_	1–4	Gamma and FN CSs increased	[151]
40Na_2_O–(60–*x*)P2O5–*x*GeO_2_	GeO_2_	0–30	Gamma CS increased; FN CS optimised at 30 mol% of P2O5	[152]
40Na_2_O–10B_2_O_3_–(50–*x*)P_2_O_5_–*x*GeO_2_	GeO_2_	0–30	Gamma CS increased; FN CS optimised at 30 mol% of P_2_O_5_	[152]
3As_2_O_3_-37PbO-(60-x)P_2_O_5_- xWO_3_	WO_3_	0–5	Gamma CS increased	[153]
45Na_2_O−10Bi_2_O_3_ − (5-x)TiO_2_ −xNb_2_O_5_−40P_2_O_5_	Nb_2_O_5_	0–5	Gamma CS increased	[154]

### 2.4 Silicate glasses

Silicate-based glass systems have been used since ancient times. They have become popular throughout human culture and civilizations. This is because the earth's crust is rich in minerals that contain silica, like quartz and sand. Silicon glasses has undergone significant transformation from natural glasses adopted more than 75 millennia ago and the discovery of modern silicate glass about 700 centuries later [154]. In recent times most silicate glasses with diverse compositions, features, and functionality have been prepared using diverse fabrication methods to meet the demand of modern technological innovations. Infact, the advancement of glass science and technology has been dependent on silicate-based glass compositions. Today, silicate-based glasses are the most common glass product with diverse applications in telecommunication, electronics, bioactive glasses used in biomedical applications, optical and window glasses, kitchen wares, and for packaging food, drinks and pharmaceutical products [[155], [156], [157]].

The choice for silicate-based glass systems has, for some reason, remained somewhat restricted in the literature when it comes to radiation shielding applications. Silicate-based glass systems can be inexpensive, easily accessible, and produced using well-established methods; however, they have low ion solubility, relatively low density values (2.5–3.5 g/cm3), and significantly high process temperatures (>1300 C) [[158], [159], [160], [161], [162], [163], [164], [165], [166], [167]]. Nevertheless, in order to comprehend their potential for radiation protection, researchers looked into a variety of silicate-based glass systems containing heavy metal oxides. A compilation of some recent studies that focused on radiation shielding parameters of silicate glasses are presented in Table 4. In some silicate glasses, fluxing agents (such Na_2_O, K_2_O, etc.) are added to lower the glass system's overall melting temperature. In addition, other oxides that create glass network, like lead or boron oxide, can be added to the silicate system to promote the creation of glass. These additions also affect the shielding behaviour of the glasses as indicated in the table. Several heavy-metal oxides, such as Ta_2_O_5_, BaO, Bi_2_O_3_, La_2_O_3_, Er_2_O_3_, and Pr_2_O_3_, when doped in different proportions, mostly within 0–10 mol% generally improve the shielding ability of silicate glasses. However, the improvement in the shielding efficacy of silicate glasses is not limited to HMOs only, less dense metal oxides including ZnO [168,169] have shown capacity to improve the shielding behaviour of silicate glasses. In a binary glass system, it is easier to see that the concentration of the heavier oxide dictate the trend of the gamma shielding quality. For example, in the glass composition (100-x)SiO_2_-xMgO, increasing the concentration of MgO relative to SiO_2_ weakens the ability of the glass to attenuate photons [170]**.** This is due to the higher linear attenuation coefficient of SiO_2_ in contrast to MgO (see Table 6, Table 8. However, in a multicomponent silicate glass system, the presence of other chemical groups might make the prediction gamma attenuation trend when certain oxides is replaced with another difficult. A case study is the phosphate glass system xAl_2_O_3_·(40−x)Ag_2_O·60P_2_O_5_ whose gamma cross-section increased when Al_2_O_3_ displaced Ag_2_O in the chemical structure [149]. This clearly shows that the gamma shielding behavior of a glass cannot be absolutely predicted by looking at the chemical structure alone, the amount of each chemical unit and photon energy of interest also needs to be considered. Naturally, the primary goal is to make glass systems more capable of attenuating high photon energy. Almost all of the investigations that looked at radiation shielding qualities revealed that the protection qualities were significantly improved, and some of the glass systems were also determined to be excellent candidates for use as radiation shielding glass in commercial settings. In summary, silicate-based glass systems show a lot of promise for low-photon energy applications, particularly when considering their inexpensive production cost and route.

**Table 4:** Summary of effects of some modifying oxides on some recently investigated silicate glass system.

Glass composition	Modifying oxide/material	Oxide concentration range (mol%)	Effect on shielding capacity	Ref.
37.5Na_2_O + (61.1 - x)SiO_2_ + xY_2_O_3_	Y_2_O_3_	1–6	Gamma CS increased; FN CS decreases	[158]
xTa_2_O_5_+(50 - x)BaO+25B_2_O_3_+15SiO_2_+10CaO	Ta_2_O_5_	0-15 wt%	Gamma CS increased saliently	[159]
35Pb_3_O_4_ + 60SiO_2_ + (5 - x)ZnO + xWO_3_	WO_3_	1–5	Gamma and FN CSs increased	[160]
(55 - x)SiO_2_ + 13B_2_O_3_ + 1Al_2_O_3_ + 4.5BaO + 6.3CaO + 0.2Sb_2_O_3_ + 20Na_2_O + xPr_2_O_3_	Pr_2_O_3_	0.5–3	Gamma CS increased	[161]
45P_2_O_5_-15B_2_O_3_-22Na_2_O-(18-x)K_2_O: xSrO	SrO	0–12	Gamma CS increased	[162]
60Bi_2_O_3_-(40-x) B_2_O_3_-xSiO_2_	B_2_O_3_	0–40	Gamma CS decreased	[163]
5Bi_2_O_3_–15SiO_2_–15TeO_2_–(55 − *x*)B_2_O_3_–*x*CeO_2_	CeO_2_	0–20	Gamma CS increased	[164]
30PbO-20SiO_2_-(50—y)Na_2_B_4_O_7_ -xTiO_2_	TiO_2_	0–45	Gamma CS increased; FN CS decreases	[165]
10Na_2_O–15PbO–10SiO_2_-(65-x)B_2_O_3_-xBaO	BaO	0–5	Gamma CS increased	[166]
(73.2SiO_2_ -15.3Na_2_O-6MgO-2ZnO- 3.5CaO)*1-x-*(TbF_3_)*x*	TbF_3_	0-15 wt%	Gamma CS increased	[167]
SiO_2_-PbO-Na_2_O- B_2_O_3_	PbO	5–15	Gamma CS increased	[168]
(SiO_2_)_20_(B_2_O_3_)_80-x_(ZnO)x	ZnO	60–67	Gamma CS increased	[169]

**Table 5:** Radiation shielding trend of some recently investigated tellurite glass system.

Glass composition	Modifying oxide/material	Oxide concentration range (mol%)	Effect on shielding capacity	Ref.
*x*Bi_2_O_3_-(80-*x*)B_2_O_3_-5TeO_2_-15SiO_2_	Bi_2_O_3_	50-75 wt%	Gamma CS increased	[171]
(60 − *x*)B_2_O_3_–(10 + *x*)TeO_2_–10ZnO–10Al_2_O_3_–5Li_2_O–5MgO	B_2_O_3_	10–60	Gamma decreased	[172]
20WO_3_-x Bi_2_O_3_- (80-x)TeO_2_	Bi_2_O_3_	10–25	Gamma CS increased	[173]
(TeO2)0.7 (B2O3)0.3]1–x (Bi2O3) x	Bi_2_O_3_	0–30	Gamma CS increased	[174]
(70-*x*)TeO_2_–10GeO_2_–10ZnO–10Li_2_O–*x*Bi_2_O_3_,	Bi_2_O_3_	0–15	Gamma CS increased	[175]
68TeO_2_‒(22-x)Bi_2_O_3_‒10ZnO‒ (x)PbO	PbO	10–18	Gamma CS decreased	[176]
(60-x)TeO_2_–10GeO_2_-10ZnO–10Li2O–10Bi_2_O_3_- B_2_O_3_	B_2_O_3_	0–25	Gamma CS decreased	[177]
(25ZnO.75TeO_2_)_100-x._(Ta_2_O_5_)_x_	Ta_2_O_5_	0-3 wt%	Gamma, CR, and FN CSs increased	[178]
90-x) TeO_2_ - 10 ZnO - xBaO	BaO	25–35	Gamma CS increased	[179]
(80-x)TeO_2_-xB_2_O_3_–5ZnO–5Li_2_O_3_–10Bi_2_O_3_	B_2_O_3_	30–80	Gamma CS decreased	[180]
74.75TeO2.0.25V2O5.(25-x)B2O3.xSm2O3	Sm_2_O_3_	0–1.5	Gamma CS increased	[181]
(25-x)ZnO–24B_2_O_3_–51TeO_2_-xEu_2_O_3_	Eu_2_O_3_	0–3	Gamma CS increased	[182]
50TeO_2_–30B_2_O_3_-(20-x) Li_2_O-xCeO_2_	CeO_2_	0–20	Gamma CS increased	[183]

**Table 6:** MAC of common glass forming oxides.

MAC (cm^2^/g)	Glass forming oxide				
	B_2_O_3_	GeO_2_	P_2_O_5_	SiO_2_	TeO_2_
0.01	4.494	27.79	20.97	19.0100	121.2
0.015	1.416	64.06	6.441	5.8090	40.96
0.02	0.6902	29.57	2.824	2.5470	18.89
0.03	0.3246	9.729	0.9549	0.8726	6.375
0.04	0.2339	4.387	0.499	0.4654	16.56
0.05	0.1987	2.38	0.3348	0.3185	9.197
0.06	0.1807	1.462	0.26	0.2515	5.667
0.08	0.1614	0.7108	0.196	0.1935	2.636
0.1	0.1502	0.4327	0.1688	0.1684	1.471
0.15	0.1324	0.2145	0.1392	0.1402	0.5465
0.2	0.1206	0.1531	0.1243	0.1255	0.2989
0.3	0.1044	0.1112	0.1063	0.1075	0.1557
0.4	0.09339	0.094	0.09476	0.0959	0.1122
0.5	0.08523	0.0837	0.08634	0.0874	0.09208
0.6	0.07881	0.07641	0.07976	0.0807	0.08032
0.8	0.0692	0.06628	0.06998	0.0709	0.06648
1	0.06222	0.05924	0.06289	0.0637	0.0581
1.022	0.06156	0.05858	0.06222	0.0630	0.05736
1.25	0.05564	0.05283	0.05623	0.0569	0.05113
1.5	0.05063	0.04819	0.05121	0.0518	0.04652
2	0.0435	0.042	0.04415	0.0447	0.04099
2.044	0.04299	0.04158	0.04365	0.0442	0.04065
3	0.035	0.03546	0.03594	0.0364	0.03604
4	0.03006	0.03221	0.03131	0.0317	0.03415
5	0.02684	0.03042	0.02837	0.0287	0.03348
6	0.02458	0.02937	0.02637	0.0266	0.03337
7	0.02292	0.02879	0.02495	0.0252	0.03358
8	0.02164	0.02846	0.02389	0.0241	0.03398
9	0.02065	0.02833	0.0231	0.0233	0.03449
10	0.01985	0.02829	0.02248	0.0226	0.03505

**Table 7:** MACs of common alkali metal oxides in glass systems.

MAC (cm^2^/g)	Alkali metal oxide				
	Li_2_O	Na_2_O	K_2_O	Rb_2_O	Cs_2_O
0.01	3.345	13.09	66.65	53.24	169.5
0.015	1.084	3.956	21.08	17.62	57.7
0.02	0.5495	1.75	9.223	54.75	26.67
0.03	0.2787	0.6315	2.897	18.4	8.99
0.04	0.2105	0.3612	1.323	8.352	22.47
0.05	0.1833	0.263	0.7566	4.516	12.65
0.06	0.1689	0.2175	0.5038	2.74	7.792
0.08	0.1528	0.1766	0.2984	1.272	3.628
0.1	0.1429	0.1576	0.221	0.7265	2.013
0.15	0.1266	0.1342	0.1544	0.3031	0.7236
0.2	0.1155	0.1209	0.1305	0.1899	0.3787
0.3	0.1001	0.104	0.1078	0.1207	0.1817
0.4	0.08954	0.09284	0.09507	0.09661	0.124
0.5	0.08173	0.08464	0.08622	0.08392	0.09845
0.6	0.07558	0.07822	0.07947	0.07558	0.08411
0.8	0.06638	0.06865	0.06956	0.0647	0.06806
1	0.05968	0.0617	0.06243	0.05747	0.05883
1.022	0.05904	0.06103	0.06176	0.05681	0.05803
1.25	0.05337	0.05517	0.0558	0.05108	0.05142
1.5	0.04856	0.05024	0.05088	0.04662	0.04672
2	0.04167	0.04328	0.0441	0.04095	0.0414
2.044	0.04117	0.04278	0.04363	0.04059	0.04109
3	0.03339	0.03516	0.03654	0.0355	0.03718
4	0.02855	0.03054	0.03251	0.03315	0.03595
5	0.02535	0.02759	0.03007	0.0321	0.03586
6	0.0231	0.02557	0.02853	0.03169	0.03625
7	0.02142	0.02412	0.02751	0.03165	0.03691
8	0.02013	0.02304	0.0268	0.03181	0.0377
9	0.01911	0.02222	0.02633	0.03212	0.03856
10	0.01829	0.02157	0.026	0.03249	0.03945

**Table 8:** MACs of some alkali earth metal oxides found in different glass systems.

MAC (cm2/g)	Alkali earth metal oxide			
	MgO	CaO	SrO	BaO
0.01	15.06	68.46	53.97	167.20
0.015	4.56	21.82	17.86	57.03
0.02	2.01	9.581	54.13	26.40
0.03	0.71	3.023	18.29	8.91
0.04	0.40	1.382	8.34	22.03
0.05	0.28	0.7895	4.519	12.37
0.06	0.23	0.5246	2.747	7.64
0.08	0.18	0.3091	1.278	3.57
0.1	0.16	0.228	0.7313	1.98
0.15	0.14	0.1584	0.3059	0.72
0.2	0.12	0.1336	0.1918	0.38
0.3	0.11	0.1103	0.122	0.18
0.4	0.10	0.09721	0.09773	0.12
0.5	0.09	0.08816	0.08488	0.10
0.6	0.08	0.08126	0.07647	0.08
0.8	0.07	0.07112	0.06546	0.07
1	0.06	0.06384	0.05815	0.06
1.022	0.06	0.06315	0.05748	0.06
1.25	0.06	0.05706	0.05168	0.05
1.5	0.05	0.05201	0.04716	0.05
2	0.04	0.04506	0.04138	0.04
2.044	0.04	0.04458	0.041	0.04
3	0.04	0.03728	0.03573	0.04
4	0.03	0.03311	0.03324	0.04
5	0.03	0.03058	0.03208	0.04
6	0.03	0.02897	0.03157	0.04
7	0.02	0.02788	0.03145	0.04
8	0.02	0.02713	0.03154	0.04
9	0.02	0.02661	0.03178	0.04
10	0.02	0.02625	0.0321	0.04

Commercial soda-lime-silica glass is known to have a density of approximately 2.5 g/cm3, and adding high-density oxides to the glass will ultimately increase its density and have a favorable effect. The most significant finding is that even higher density values can be attained without the use of lead oxide content, which is essential for getting rid of lead and its derivatives because they are poisonous. All things considered, silicate-based glass systems can meet the requirements of radiation shielding applications, especially those involving low energy.

### 2.5 Telluride glasses

Especially in recent years, TeO_2_-based glasses have attracted much attention from the scientific community. They have become prominent, particularly in photonics, optoelectronics, and optical fibers applications, concerning their unique optical properties and other material aspects. In addition, telluride glass can be processed at considerably lower temperatures, which is essential for energy-saving attempts in the glass manufacturing industry. TeO_2_ has a high-density value (5.67 g cm^−3^), wide band gap (0.4–6.0 μm), high refractive index (>2.25), melting at low temperatures (700–800 °C), enabling high ion solubility and providing good mechanical resistance are its main properties. Nowadays, telluride glass systems have been investigated in terms of radiation protection properties because they have high-density values without lead-oxide addition. As is known, the present motivation is to eliminate lead oxide from glass composition to attain environmentally-friendly glass systems within radiation shielding applications. Therefore, the combination of many benefits of telluride glass systems has now canalized researchers to study a variety of compositions. Theoretically and practically, TeO_2_ cannot singly form a glassy structure, instead, it requires other glass-forming agents to constitute a binary system. Boron oxide, B_2_O_3_, has come to the forefront among the glass-forming agents due to its low-melting temperatures, excellent glass-forming ability, high thermal resistance, and good mechanical properties. Further, silicon oxide, SiO_2_, can sometimes be preferred to constitute a silica-telluride system. With this in mind, other high-density oxides, including bismuth oxide, barium oxide, zinc oxide, gadolinium oxide, and the likes have been added to facilitate the properties, specifically for radiation shielding applications. In the literature, radiation shielding researchers have synthesized and investigated different types of telluride-based glass systems. Some recent investigations with their details are summarized in Table 5.

These studies highlight the function of various oxides in improving the radiation attenuation a capacity of tellurite glasses. In particular, heavy metal oxides including BaO, Bi_2_O_3_, WO_3_, PbO etc. [[171], [172], [173], [174]] improves the gamma and CR shielding efficacy of tellurite glasses.

## 3 Glass composition and shielding efficacy

Results from all investigated glasses for radiation attenuation properties indicated that chemical structure influenced the radiation protection potentials of a glass medium significantly. When there is an interplay between increasing one chemical units or species in a glass system at the expense of another, their individual shielding characteristics, densities, and molar masses are the factors that determine how their concentrations influence the radiation shielding capacity of the glass system. In order to buttress this assertion, the photon and neutron interaction parameters of some known glass formers and modifying oxides (heavy metals oxides (HMOs), alkali oxides (AEOs), alkali oxides (AO), transition metal oxides (TMOs), rare earth metal oxides (REMOs) are presented in Table 6, Table 7, Table 8, Table 9, Table 10, Table 11, Table 12, respectively. From the values of the mass attenuation coefficients (MAC), it can be hypothetically inferred how the chemical units that makeup a glass system influences its overall gamma shielding ability. The mean atomic number <Z> (Table 12) was estimated using the number of atoms in the formula unit ($n_{i}$) and $n=\sum n_{i}$ as:

(16) $$<Z>=\frac{1}{n}\sum n_{i}Z_{i}$$

**Table 9:** MACs of some transition metal oxides found in different glass systems.

MAC (cm^2^/g)	Alkali metal oxide						
	TiO_2_	V_2_O_5_	Cr_2_O_3_	Fe_2_O_3_	NiO	CuO	ZnO
0.01	68.72	70.82	96.8	121.20	165.5	173.7	188.4
0.015	22.24	23.11	31.86	40.48	56.04	59.53	65.58
0.02	9.85	10.28	14.22	18.22	25.49	27.18	30.05
0.03	3.13	3.283	4.521	5.83	8.209	8.794	9.769
0.04	1.43	1.499	2.035	2.62	3.67	3.936	4.377
0.05	0.81	0.8486	1.129	1.43	1.99	2.13	2.366
0.06	0.54	0.5565	0.7198	0.90	1.228	1.311	1.452
0.08	0.31	0.3187	0.3886	0.47	0.61	0.6433	0.705
0.1	0.23	0.2294	0.2656	0.31	0.3821	0.3975	0.4301
0.15	0.15	0.1541	0.1653	0.18	0.2026	0.2045	0.2148
0.2	0.13	0.1282	0.1333	0.14	0.1508	0.1494	0.1542
0.3	0.11	0.1049	0.1068	0.11	0.1136	0.1109	0.1127
0.4	0.09	0.0923	0.09324	0.09	0.09722	0.09444	0.09544
0.5	0.08	0.08362	0.08423	0.09	0.08705	0.08436	0.08505
0.6	0.08	0.07702	0.07747	0.08	0.07971	0.07715	0.07769
0.8	0.07	0.06739	0.06768	0.07	0.06933	0.06702	0.06741
1	0.06	0.06048	0.06069	0.06	0.06205	0.05995	0.06026
1.022	0.06	0.05983	0.06003	0.06	0.06136	0.05929	0.05959
1.25	0.05	0.05405	0.05422	0.05	0.05538	0.05349	0.05374
1.5	0.05	0.04926	0.04943	0.05	0.05052	0.0488	0.04903
2	0.04	0.04266	0.04291	0.04	0.04403	0.04256	0.04279
2.044	0.04	0.0422	0.04246	0.04	0.04358	0.04214	0.04237
3	0.04	0.03523	0.03571	0.04	0.03713	0.03598	0.03627
4	0.03	0.03123	0.03192	0.03	0.0337	0.03274	0.03309
5	0.03	0.0288	0.02969	0.03	0.03179	0.03096	0.03137
6	0.03	0.02722	0.02828	0.03	0.03069	0.02996	0.03041
7	0.03	0.02615	0.02738	0.03	0.03006	0.0294	0.0299
8	0.03	0.02541	0.02677	0.03	0.02971	0.02911	0.02966
9	0.03	0.02489	0.02639	0.03	0.02955	0.02899	0.02958
10	0.02	0.02452	0.02613	0.03	0.0295	0.02899	0.02962

**Table 10:** MACs of some rare earth metal oxides found in different glass systems.

MAC (cm2/g)	Alkali metal oxide							
	CeO_2_	Sm_2_O_3_	Eu_2_O_3_	Gd_2_O_3_	Dy_2_O_3_	Ho_2_O_3_	Er_2_O_3_	La_2_O_3_
0.01	170.60	216.4	227.9	234.40	253.6	263.7	274.4	168.6
0.015	58.48	74.69	78.74	81.23	88.8	92.81	96.97	57.67
0.02	27.13	34.83	36.75	37.95	41.62	43.59	45.61	26.72
0.03	9.18	11.83	12.5	12.92	14.21	14.9	15.61	9.029
0.04	4.29	5.522	5.837	6.04	6.639	6.963	7.304	22.02
0.05	4.17	3.661	3.529	3.38	3.71	3.891	4.079	12.37
0.06	7.73	9.574	10	10.22	10.99	11.45	11.93	7.669
0.08	3.62	4.517	4.735	4.86	5.262	5.47	5.687	3.586
0.1	2.02	2.524	2.647	2.72	2.948	3.069	3.192	1.997
0.15	0.73	0.9043	0.9478	0.97	1.053	1.096	1.141	0.7226
0.2	0.39	0.4648	0.4855	0.50	0.5346	0.5549	0.5765	0.3797
0.3	0.19	0.2127	0.2202	0.22	0.2367	0.2439	0.2516	0.183
0.4	0.13	0.1396	0.1434	0.14	0.1508	0.1544	0.1582	0.1251
0.5	0.10	0.1079	0.1103	0.11	0.1142	0.1162	0.1185	0.09941
0.6	0.09	0.0906	0.09222	0.09	0.09442	0.0958	0.0973	0.08496
0.8	0.07	0.07185	0.07282	0.07	0.07354	0.07428	0.07508	0.06879
1	0.06	0.06143	0.06211	0.06	0.06225	0.06272	0.06324	0.05949
1.022	0.06	0.06052	0.06119	0.06	0.06131	0.06174	0.06225	0.05866
1.25	0.05	0.05329	0.05381	0.05	0.05366	0.05394	0.0543	0.05196
1.5	0.05	0.04829	0.04872	0.05	0.0485	0.04874	0.04901	0.04721
2	0.04	0.0427	0.04309	0.04	0.0429	0.04311	0.04335	0.04172
2.044	0.04	0.04238	0.04276	0.04	0.04257	0.04278	0.04302	0.04139
3	0.04	0.03831	0.0387	0.04	0.03865	0.03888	0.03913	0.03716
4	0.04	0.03701	0.03743	0.04	0.03752	0.03778	0.03806	0.03566
5	0.04	0.03688	0.03734	0.04	0.03752	0.03781	0.03812	0.03534
6	0.04	0.03725	0.03774	0.04	0.03801	0.03833	0.03867	0.03553
7	0.04	0.03789	0.03842	0.04	0.03876	0.0391	0.03947	0.03601
8	0.04	0.03868	0.03923	0.04	0.03963	0.04001	0.0404	0.03665
9	0.0373	0.03953	0.04012	0.03988	0.04058	0.04097	0.04139	0.03738
10	0.03803	0.04043	0.04104	0.04081	0.04155	0.04197	0.0424	0.03815

**Table 11:** MACs of some heavy metal oxides found in different glass systems.

MAC (cm^2^/g)	Alkali metal oxide					
	PbO	Bi_2_O_3_	WO_3_	Sb_2_O_3_	CdO	MoO_3_
0.01	121.70	122.6	78.07	122.80	109.6	59.14
0.015	103.70	104.2	110.5	41.43	36.8	19.63
0.02	80.24	80.38	52.29	19.09	16.92	53.31
0.03	28.17	28.31	18.1	6.44	33.01	18.85
0.04	13.35	13.44	8.514	16.97	15.59	8.711
0.05	7.48	7.537	4.762	9.39	8.586	4.763
0.06	4.67	4.713	2.984	5.77	5.256	2.912
0.08	2.26	2.279	6.227	2.68	2.429	1.364
0.1	5.16	5.162	3.55	1.49	1.353	0.7821
0.15	1.88	1.882	1.282	0.55	0.5066	0.3259
0.2	0.94	0.9396	0.6477	0.30	0.2814	0.2028
0.3	0.38	0.3846	0.2789	0.16	0.1508	0.1276
0.4	0.22	0.2244	0.1724	0.11	0.1107	0.1017
0.5	0.16	0.1575	0.1273	0.09	0.09185	0.08808
0.6	0.12	0.1228	0.1034	0.08	0.08065	0.07924
0.8	0.09	0.08836	0.07864	0.07	0.06722	0.06775
1	0.07	0.07127	0.06567	0.06	0.05894	0.06015
1.022	0.07	0.06989	0.06457	0.06	0.0582	0.05945
1.25	0.06	0.05927	0.05601	0.05	0.05199	0.05343
1.5	0.05	0.05275	0.05038	0.05	0.04737	0.04871
2	0.05	0.04638	0.04438	0.04	0.04179	0.04262
2.044	0.05	0.04603	0.04404	0.04	0.04144	0.04222
3	0.04	0.04209	0.03976	0.04	0.03686	0.03649
4	0.04	0.04124	0.03843	0.03	0.03502	0.03364
5	0.04	0.04159	0.03829	0.03	0.03442	0.03219
6	0.04	0.04243	0.03867	0.03	0.03437	0.03144
7	0.04	0.04353	0.03934	0.03	0.03466	0.03111
8	0.05	0.04472	0.04015	0.04	0.03513	0.03103
9	0.04633	0.04597	0.04104	0.03557	0.0357	0.03111
10	0.04765	0.04723	0.04197	0.03619	0.03633	0.03129

**Table 12:** Density, molar weight, mean Z, and CSs fr thermal neutrons of common oxides in glasses.

Glass oxides	Density (g/cm^3^)	<Z>	Molar mass	Thermal neutron CS (cm^−1^)		
				Σ_sc_	Σ_ab_	Σ_tot_
B_2_O_3_	2.46	6.8	69.617	0.22295	32.63186	32.85481
Ge_2_O_3_	5.97	17.6	193.997	0.31865	0.08152	0.40018
P_2_O_5_	2.39	10.8	141.995	0.06713	0.00350	0.07062
SiO_2_	2.196	10	59.998	0.04776	0.00378	0.05153
TeO_2_	5.67	22.67	159.998	0.09217	0.10028	0.19245
Li_2_O	2.01	4.67	29.999	0.11053	5.68729	5.79782
Na_2_O	2.27	10	61.999	0.14460	0.02337	0.16796
K_2_O	2.35	15.33	93.999	0.05900	0.06321	0.12221
Rb_2_O	4	27.33	186.939	0.17519	0.00979	0.18498
Cs_2_O	4.65	39.33	281.999	0.07743	0.57575	0.65318
MgO	3.58	10	39.999	0.19991	0.00340	0.20331
CaO	3.35	14	55.999	0.10192	0.01549	0.11742
SrO	4.7	22	103.999	0.17004	0.03483	0.20487
BaO	5.72	32	152.999	0.07608	0.02476	0.10084
TiO_2_	4.26	12.67	79.998	0.13946	0.19524	0.33470
V_2_O_5_	3.36	12.29	181.995	0.11337	0.11293	0.22631
Cr_2_O_3_	5.22	14.4	151.997	0.14432	0.12613	0.27044
Fe_2_O_3_	5.24	15.2	159.997	0.45821	0.10096	0.55916
NiO	6.67	18	72.999	1.01761	0.24698	1.26460
CuO	6.4	18.5	79.999	0.38674	0.18206	0.56880
ZnO	5.61	19	80.999	0.17225	0.04629	0.21854
CeO_2_	7.65	24.67	171.998	0.07873	0.01688	0.09561
Sm_2_O_3_	8.35	29.6	347.997	1.12669	171.08262	172.20931
Eu_2_O_3_	7.4	30	351.997	0.23287	114.66146	114.89434
Gd_2_O_3_	7.41	30.4	361.997	4.43622	1224.88725	1229.32347
Dy_2_O_3_	7.8	31.2	373.997	0.00754	24.95971	24.96725
Ho_2_O_3_	8.41	31.6	377.997	0.22556	1.73317	1.95873
Er_2_O_3_	8.64	32	381.997	0.23693	4.32990	4.56683
La_2_O_3_	6.51	27.6	325.997	0.23227	0.21568	0.44794
PbO	9.53	45	222.999	0.28604	0.00440	0.29044
Bi_2_O_3_	8.9	38	465.997	0.21055	0.00078	0.21133
WO_3_	7.16	24.5	231.997	0.08547	0.34001	0.42548
Sb_2_O_3_	5.2	25.2	291.997	0.08363	0.10528	0.18891
CdO	8.15	28	127.999	0.24916	96.59354	96.84270
MoO_3_	4.69	16.5	143.997	0.11197	0.04864	0.16061

Σ_sc_ = thermal neutron scattering cross-section, Σ_ab_ = thermal neutron absorption cross-section, Σ_tot_ = Σ_sc_+ Σ_ab_ = thermal neutron total cross-section.

Since the interaction of gamma radiation is mostly with orbital electrons, the mean atomic number could be used to ascertain how many electron is presented by an oxide for photon interaction. The number could be used as a rough estimate for comparing attenuation prowess. In Table 6, the MAC values of glass forming oxides are presented. For most of the energy spectrum, the trend of MAC is consistent with molecular weight, mean atomic number (<Z>), and density of the oxides. Thus a denser oxide with higher mean electrons would attenuate photon better. The denser oxide would also, improve the shielding behaviour of a glass system when the concentration is increased. In 10SrO-(90-x)B_2_O_3_-xTeO_2_ [113] and 40Na_2_O–10B_2_O_3_–(50–*x*)P_2_O_5_–*x*GeO2 [152] systems, the partial replacement of B_2_O_3_ and P_2_O_5_ with TeO_2_ and GeO_2_, respectively, resulted in the increase of the attenuation coefficients of the glasses due to the higher density and <Z> of the replacing oxides. The high density, <Z>, and ultimately the MAC of heavy metal oxides such as TeO_2_, BaO, CdO, WO_3_, PbO etc often improved the gamma shielding properties of glasses as seen in many glass systems [[116], [117], [118], [119], [120], [121], [122], [123], [124], [125], [126], [127], [128], [129], [130], [131], [132], [133]]. However, it must be noted that the introduction, doping, or relative increase of denser oxides or ions into a glass system does not necessarily produce an increase in the gamma attenuation coefficients. The effect depends on the level of increment and how the increment affects the weight fraction of other chemical units within the glass system. In the (40-x)PbO–50B_2_O_3_–xBi_2_O_3_–10ZnO [121] glass system, despite the high density of Bi_2_O_3_ and similar gamma shielding coefficient as PbO (as seen in Table 11), a partial replacement of PbO with Bi2O3 caused a decline in the ability of the glass to absorb gamma photons. Similarly, in Table 5, when their roles of PbO and Bi_2_O_3_ were reversed in 68TeO_2_‒(22-x)Bi_2_O_3_‒10ZnO‒ (x)PbO [177], despite the higher MAC of PbO, the MAC of the glass system decreased. This anomaly is often observed when the relative density or <Z> of the one oxide/unit is close to the replacing units. In addition, the amount of replacement must be significant in some cases before an increment is observed. Increasing one unit relative to another may not be feasible due to other glass properties, hence compensation is made for glass thickness to make up for the reduction in shielding behavior. The reaction of the shielding behavior of a glass medium to changes in the chemical composition thus depends on the distributions of the chemical constituents in terms of their weight fractions, mass attenuation coefficients and range of variation. These parameters are summarized in the additive equation of MAC as follows [8]:

(17) $$MAC=\sum {MAC}_{i}w_{i}$$

Hence, for binary variations, the weight fraction (w) and MAC of the two chemical units changing with respect to one another and the weight fractions of the other heavy oxides in the glass are important factors.

For neutrons, interaction is with individual atoms, hence the CSs of all the atoms present within the glass system will determine the shielding behaviour of the glass system. In Table 12, the CS of common chemical units in glass are presented. These data can be use to understand why neutron shielding ability changes when these units are altered in a glass system. Reducing the contents of units with high neutron CSs would reduce neutron interaction prowess of resulting glasses. For example, the relative high neutron CS of CdO compared to TeO_2_ dictated the neutron shielding behaviour of 50B_2_O_3_ - (50-x)TeO_2_- xCdO as the two oxides vary [122].

## 4 Trend in glass shield research

Aside the focus on the shielding requirements of pristine glasses, in an attempt to find alternative uses for waste glasses, some recent studies have also focused on the upcycling of waste glasses for shielding applications. Waste cathode-ray tube (CRT) glasses have be upcycled to effective shielding glasses by doping with different materials including Bi_2_O_3_, Li_2_O, Y_2_O_3_, Er, Na_2_O, and CoO [[184], [185], [186], [187], [188], [189], [190]]**.** Also, waste car window, mobile phone, soda lime, borosilicate glasses have all been prepared with enhanced radiation shielding attributes by doping with PbO, Bi_2_O_3_, BaO, SrO, Ta_2_O_5_, WO_3_, and Y_2_O_3_ [[191], [192], [193], [194], [195], [196], [197]]**.** These glasses showed potential to outperform some pristine shielding glasses and conventional shields. This clearly showed that radiation shielding glasses could be produced from raw materials or from waste. The production of shielding glasses from waste is important for resource management and environmental conservation. Another inference from these studies is that produced pristine glass shields could be used in a closed-loop recycling to produce new shielding glasses [[198], [199], [200], [201], [202], [203], [204], [205]]. This is important from economic perspective. Therefore, further research on the use of waste glasses for shielding functions will increase in the future.

No doubt there are many research focused on the shielding properties of glasses. There has also been consistent increase in such research due to the expanding application of radiation, drawback of some glass systems to function in certain radiation environments, need to improve shielding competency of existing glasses, and producing novel shielding materials. Many of the existing research have similar focus, which is the determination of shielding attributes only. In the future, there would be the need to study other properties of the glasses that makes them suitable for shielding in specific areas of radiation applications. Also, the long term usage of the glasses needs to be studied by highlighting radiation damage in them after irradiation with specific radiation for a long time. The manner in which the damage will affect the glass shielding behavior is also a major factor for future investigation. Long term usage of glasses would also depend on activation after irradiation. Consequently, glass components that can be activated by certain radiation and doses which makes the shield a source of radiation must be avoided when designing shields. Although several factors needs to be considered in making a choice of material for shielding, glasses can be tailored to fulfilled the requirements in all shielding scenarios. The flexibility of glassy materials along with their properties will continue to bring glasses in the forefront of radiation shielding research and parameters. With proper research and deligence, glass shields is the future of radiation control.

## 5 Conclusion

The application of radiation will continue to expand so also will be the research and deployment of shielding materials. At present, many shielding materials are available but no one fits all shields. Conversely different shielding materials are suited for different radiation environments. Borate, germanate, silicate, phosphate and tellurite glasses are mostly investigated for shielding applications. Germanate glasses are mostly used for optical applications while borate are silicate glasses are mostly adopted for everyday glass applications due to the abundance of silicate-based natural material. The chemical composition of glass influence their shielding performance greatly, while heavy metal content of glasses improves the gamma radiation absorption. Silicate-based glass systems show a lot of promise for low-photon energy applications, particularly when considering their inexpensive production costs. Their shielding characteristics can be improved by using costly HMO. Tellurite glasses and other glass systems containing heavy elements and having moderate to high density a desireable and more effective for gamma attenuation purposes. Neutron shielding however require light elements and heavy elements such as Cd, Sm, Gd etc with high neutron cross section. A good mix of chemical elements will ensure good shielding quality in glasses. Although many heavy metal improve gamma shielding, the scarcity of the elements will prevent the wide spread use of glasses containing them for shielding application. The flexibility of glassy materials along with their properties will continue to bring glasses in the forefront of radiation shielding research and parameters. With proper research and deligence, glass shields is the future of radiation control. Future research in the shielding behaviour of pristine and waste glasses would not only state the shielding parameters of the glasses but radiation impact and stability of the glasses in radiation environments.

## CRediT authorship contribution statement

**M.S. Al-Buriahi:** Writing – review & editing, Conceptualization. **Recep Kurtulus:** Formal analysis, Data curation. **Canel Eke:** Investigation, Data curation. **Sultan Alomairy:** Supervision and funding. **I.O. Olarinoye:** Writing – review & editing.

## Declaration of competing interest

The authors declare that they have no known competing financial interests or personal relationships that could have appeared to influence the work reported in this paper.
